# Protection of Macaques with Diverse MHC Genotypes against a Heterologous SIV by Vaccination with a Deglycosylated Live-Attenuated SIV

**DOI:** 10.1371/journal.pone.0011678

**Published:** 2010-07-20

**Authors:** Chie Sugimoto, Satoru Watanabe, Taeko Naruse, Eiji Kajiwara, Teiichiro Shiino, Natsuko Umano, Kayoko Ueda, Hirotaka Sato, Shinji Ohgimoto, Vanessa Hirsch, Francois Villinger, Aftab A. Ansari, Akinori Kimura, Masaaki Miyazawa, Yasuo Suzuki, Naoki Yamamoto, Yoshiyuki Nagai, Kazuyasu Mori

**Affiliations:** 1 AIDS Research Center, National Institute of Infectious Diseases, Shinjuku-ku, Tokyo, Japan; 2 Tsukuba Primate Research Center, National Institute of Biomedical Innovation, Tsukuba, Ibaraki, Japan; 3 CREST, Japan Science and Technology Agency, Kawaguchi, Saitama, Japan; 4 Division of Medical Science, Department of Molecular Pathogenesis, Medical Research Institute, Tokyo Medical and Dental University, Chiyoda-ku, Tokyo, Japan; 5 Department of Immunology, Kinki University School of Medicine, Osaka-Sayama, Osaka, Japan; 6 Department of Virology, Osaka City University Graduate School of Medicine, Abeno-ku, Osaka, Japan; 7 Laboratory of Molecular Microbiology, National Institute of Allergy and Infectious Diseases, National Institutes of Health, Bethesda, Maryland, United States of America; 8 Department of Pathology and Laboratory Medicine, Emory University, Atlanta, Georgia, United States of America; 9 Yerkes National Primate Research Center, Emory University, Atlanta, Georgia, United States of America; 10 Department of Biomedical Sciences, College of Life and Health Sciences, Chubu University, Kasugai, Aichi, Japan; 11 Center of Research Network for Infectious Diseases, Riken, Chiyoda-ku, Tokyo Japan; University of California San Francisco, United States of America

## Abstract

HIV vaccine development has been hampered by issues such as undefined correlates of protection and extensive diversity of HIV. We addressed these issues using a previously established SIV-macaque model in which SIV mutants with deletions of multiple gp120 *N*-glycans function as potent live attenuated vaccines to induce near-sterile immunity against the parental pathogenic SIVmac239. In this study, we investigated the protective efficacy of these mutants against a highly pathogenic heterologous SIVsmE543-3 delivered intravenously to rhesus macaques with diverse MHC genotypes. All 11 vaccinated macaques contained the acute-phase infection with blood viral loads below the level of detection between 4 and 10 weeks postchallenge (pc), following a transient but marginal peak of viral replication at 2 weeks in only half of the challenged animals. In the chronic phase, seven vaccinees contained viral replication for over 80 weeks pc, while four did not. Neutralizing antibodies against challenge virus were not detected. Although overall levels of SIV specific T cell responses did not correlate with containment of acute and chronic viral replication, a critical role of cellular responses in the containment of viral replication was suggested. Emergence of viruses with altered fitness due to recombination between the vaccine and challenge viruses and increased gp120 glycosylation was linked to the failure to control SIV. These results demonstrate the induction of effective protective immune responses in a significant number of animals against heterologous virus by infection with deglycosylated attenuated SIV mutants in macaques with highly diverse MHC background. These findings suggest that broad HIV cross clade protection is possible, even in hosts with diverse genetic backgrounds. In summary, results of this study indicate that deglycosylated live-attenuated vaccines may provide a platform for the elucidation of correlates of protection needed for a successful HIV vaccine against diverse isolates.

## Introduction

Molecular epidemiological studies have revealed the existence of an extensive degree of diversity of HIV-1 isolates [1]. HIV-1 is classified in three major groups (M, N, O) based on their geographical origin. Group M represents the predominant HIV-1 circulating through the world and has been divided into more than 10 subtypes (clades) as well as increasing number of circulating recombinant forms (CRF) primarily due to error-prone viral reverse transcriptase and the occurrence of super-infections. This diversity is continuously expanding worldwide and is a major obstacle for the successful development of an AIDS vaccine. While the generation of a vaccine capable to prevent transmission of HIV isolates endemic in a particular area remains an unfulfilled task, protection against phylogenetically distant viruses represents an even more formidable hurdle. The failure and dismal success of HIV-1 vaccine trials that have been conducted so far has prompted a re-emphasis for more basic studies concerning vaccine design against heterologous challenge viruses, which can at present only be addressed in a macaque model. One of the pre-conditions for the objective assessment of the protective efficacy against a heterologous strain would be that the macaque model used should have the capacity to confer sterile or near-sterile immunity against the homologous virus challenge.

SIVmac239 infected rhesus macaques gradually develop AIDS after a variable period of chronic infection. In order to investigate the role and function of the glycan shield of the viral envelope, we previously developed a panel of deglycosylated mutants from this pathogenic SIVmac239 backbone [2]. Among these mutants, one mutant with five *N*-glycans deleted (Δ5G) was found to be profoundly attenuated in rhesus macaques. Thus, while the acute primary viremia showed viral peaks undistinguishable from those measured in animals infected with the wild-type SIVmac239 infection, viral load during the chronic phase was contained at or below the level of detection [3]. More importantly, these Δ5G “immunized” macaques during the chronic phase manifested near-sterile immunity when challenged with the homologous wild-type SIVmac239, and the animals showed neither evolution of pathogenic revertants nor clinical disease manifestation during a 10 year follow up period. While it is clear that similar live attenuated HIV-1 vaccines will not likely be utilized in humans, it is extremely important to have an animal model that shows protection against heterologous challenge virus so that minimally such a model can be exploited to identify reproducible immune correlates of protection. We therefore reasoned that our SIVmac239-deglycosylation platform may provide an unique opportunity to test and analyze protection against challenge with heterologous isolates.

The studies reported herein utilized a series of four deglycosylated SIVmac239 mutants as potential live attenuated vaccine viruses and the SIVsmE543-3 isolate [4] as the heterologous challenge virus. We submit that the diversities between the vaccine viruses and the challenge virus are equivalent to those found between major HIV-1 subtypes. Thus, this heterologous challenge model provides an ideal model to assess the potential of and define the conditions for cross-subtype (clade) protection against HIV.

The natural protective effects of select rhesus macaque (Mamu) MHC class I alleles such as Mamu B*08, Mamu B*17, Mamu A*01 and the MHC class I haplotype 90120-Ia have been shown to be associated with better control of SIV [5], [6], [7], [8], [9]. In sharp contrast, protection by the deglycosylated SIV mutants exhibited no such selectivity; protection was achieved in all 9 rhesus macaques tested so far, which were later found to be indeed genetically highly diverse. Previous human cohort studies revealed that individuals who demonstrated control of HIV infection without any treatment, called long-term non-progressors and elite controllers, have common genetic properties associated with susceptibility to HIV or anti-viral host responses [10], [11], [12]. However, candidate vaccines that are aimed at targeting outbred human population will have to show effectiveness in humans with diverse genetic backgrounds. In order to minimize the contribution of particular positive or negative genetic background, macaques possessing the above described elite genotypes were therefore eliminated from the studies reported herein. Furthermore, the macaques were grouped based on the genetic data so that each group comprised animals with an essentially similar genetically diverse background.

We herein report data from a series of studies that support the concept that cross-subtype control of HIV-1 is theoretically possible irrespective of genetic background. Data derived herein demonstrate a critical role that glycosylation plays in not only conferring attenuation of SIV/HIV but also the potential role glycosylation plays in conferring pathogenic properties to viruses that emerge following challenge with heterologous viruses.

## Results

### Genetic diversity of the challenge virus from the vaccine virus

**Figure 1:**
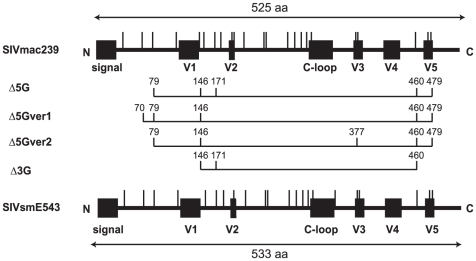
Live attenuated vaccines with deglycosylation mutations. N-glycosylation sites (vertical bars) localized within gp120 of SIVmac239 and SIVsmE543-3 are shown. SIVmac239 and SIVsmE543-3 have 23 and 22 *N*-glycosylation sites, respectively. The position of N-glycosylation sites mutated to remove the specific glycans for Δ5G, Δ5Gver-1, Δ5Gver-2, and Δ3G were indicated and constructed by site-directed mutagenesis based on SIVmac239. V1 to V5 indicate variable region 1 to 5 respectively. C-loop indicates the constant loop region within SIVmac239 [15].

SIVs are as diverse as the HIV-1 subtypes in group M, and at present a total of 9 different SIV lineages have been identified [13]. SIVmac239 belongs to lineage 8. We have generated a variety of modified candidate live vaccine strains by the introduction of deglycosylation mutations into multiple *N*-glycosylation sites of the gp120 of SIVmac239 (Fig. 1). The heterologous challenge virus used in this study is the molecularly cloned pathogenic strain SIVsmE543-3 that belongs to lineage 1. SIVmac239 and SIVsmE543-3 possess 23 and 22 *N*-glycosylation sites, respectively, and as seen their topologies in the gp120 protein backbones are almost the same (Fig. 1).

At first, we compared the amino acid sequence differences for the individual viral proteins between SIVmac239 and SIVsmE543-3 (Table 1). The genetic differences varied from 7.9% for Pol to 35.9% for Tat. We then compared the diversity between the 2 SIV strains utilized herein with the intra-subtype or inter-subtype diversities in the HIV-1 isolates and found that the differences between SIVsmE543-3 and SIVmac239 were significantly greater than any intra-subtype diversities of HIV-1 (Table 1). For the inter-subtype diversity analysis, we used subtypes B and C and a circulating recombinant CRF01_AE as reference strains that are predominantly circulating in Asian countries. The data indicated that the differences between the two SIV strains were as high or higher as those found among the three HIV-1 subtypes. These results validate the use of SIVmac239 as the parental virus for live attenuated vaccine virus and the SIVsmE543-3 as the heterologous challenge virus in the rhesus macaque model of human AIDS.

**Table 1:** Differences between the vaccine and challenge SIV and inter-subtype differences of HIV-1.

Viral proteins	SIV^b^	HIV^a^						
	mac239 vs. smE543-3	Intra-subtype (A, B, C, D, F1, G, CRF01_AE, and CRF_02AG)	Inter-subtype					
			B vs. C		B vs. CRF01		C vs. CRF01	
			Mean	S.E.	Mean	S.E.	Mean	S.E.
Gag	11.1	4.3–7.1	10.2	1.2	11	1.4	12.8	1.5
Pol	7.9	2.8–6.5	7.8	0.7	8	0.8	7.5	0.7
Env	18.2	7.7–12.4	19.2	1.4	18.8	1.4	18.7	1.4
Nef	26.2	9.0–16.2	22.6	4.8	21.3	4.8	16.2	3.5
Tat	35.9	9.9–18.1	28.8	4.7	31.9	4.9	27.4	4.3
Rev	32.7	9.0–16.5	28.3	4.7	26.4	4.7	20.6	4.1
Vif	17.8	7.0–14.2	20.5	2.6	21.6	2.9	21.3	2.8
Vpr	14.9	5.4–10.6	13.1	3.2	13	3.4	5	3.5
Vpx	8.1	NA^c^	NA		NA		NA	
Vpu	NA	2.4–14.8	17.7	5.6	3.8	5.4	12.7	3

a Percentage amino acid sequence differences per site from averaging overall sequence pairs between the subtypes.

b Percent amino acid sequence differences per protein.

c Not applicable.

### Properties of the 3 new deglycosylation mutants as live attenuated candidate vaccines

**Figure 2:**
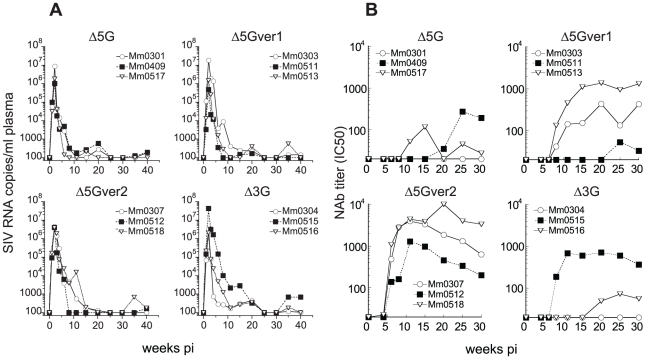
Viral loads and neutralizing antibodies in macaques infected with each of 4 deglycosylated SIV mutants. Twelve animals were divided into 4 groups consist of 3 animals each and infected with each of 4 deglycosylation mutants (Δ5G, Δ5Gver-1, Δ5Gver-2, and Δ3G). (A) Plasma viral loads were determined by real-time RT-PCR with SIVmac239 primers and probe set. (B) NAb responses against each respective infecting virus were measured in CEMx174/SIVLTR-SEAP system. NAb titers were indicated as the reciprocal of the dilutions of the plasma from the vaccinees yielding 50% inhibition (IC_50_).

We previously reported that Δ5G, a SIVmac239 molecular clone with quintuple deglycosylation mutations behaved as a live-attenuated virus in vivo [2], [3]. In addition to Δ5G, we tested three newly constructed deglycosylated mutants of SIVmac239 viruses, Δ5G-ver1, Δ5G-ver2 and Δ3G as potential candidate vaccines in this study (Fig. 1). They differ by the sites or numbers of N-glycosylation sites mutated in gp120 (Fig. 1). All four deglycosylated mutants replicated well in rhesus peripheral blood mononuclear cells (PBMC) in vitro, and the replication kinetics were similar to SIVmac239 ([2], and data not shown). However, differences were noted in the rate of replication in macrophage cultures and sensitivity to neutralizing antibodies (NAb) (data not shown). To investigate whether these differences translated into altered in vivo properties such as viral replication kinetics in rhesus macaques, reduced pathogenicity and potential vaccine properties, 12 animals were inoculated intravenously in groups of three with 100 TCID_50_ of each of the four mutants (Fig. 2 A). Since the MHC types have been shown to significantly influence the outcome of HIV/SIV infection in their respective hosts, we chose macaques which did not inherit any of the known elite MHC alleles [5], [6], [7], [8], [9] (File S1). Furthermore, to minimize the possible influence of other MHC types, we distributed the animals evenly into vaccine and control groups such that each group comprised animals with randomized MHC alleles (File S1).

**Figure 3:**
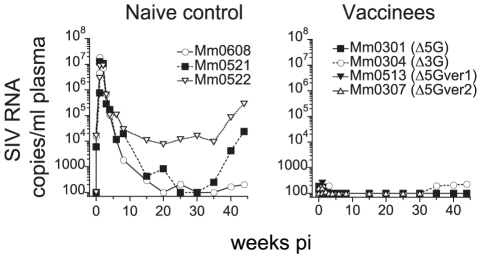
Plasma viral RNA loads in the homologous SIVmac239 challenge. Three naïve rhesus macaques (Mm0608, Mm0521, Mm0522) and 4 vaccinees (Mm0301, Mm0304, Mm0513, Mm0307), i.e. one animal from 4 deglycosylated SIV infection groups, were challenged intravenously with 1000 TCID_50_ of SIVmac239. Plasma viral loads were determined by real-time RT-PCR with SIVmac239 primers and probe set.

Consistent with our previous studies [3], the prototypic vaccine strain Δ5G, replicated as robustly as the SIVmac239 in macaques with peak plasma viral loads (VL) of ∼10^7^ copies/ml at 2 weeks post infection (pi) (Figs. 2A and 3). However, subsequently the VL of Δ5G rapidly declined to a level around or below the level of detection (100 copies/ml) whereas relatively high VL persisted in SIVmac239-infected macaques (Figs. 2A and 3). Essentially the kinetics of viremia observed with the three deglycosylation mutants, Δ5G-ver1, Δ5G-ver2 and Δ3G were similar to that seen with Δ5G (Fig. 2 A).

It has been well established that SIVmac239 elicits poor NAb in macaques [14]. In contrast, a deglycosylation mutant derived from SIVmac239 elicited higher NAb than SIVmac239, but levels of NAb responses varied among the animals [15]. Thus, we determined levels of potential NAb responses against each animal's respective infecting virus. Consistent with our previous results, most macaques infected with each of the deglycosylated SIVs induced NAb (Fig. 2 B). However, the levels of NAb responses differed among the four groups, with a decreasing order of magnitude for NAb responses from Δ5G-ver2>Δ5G-ver1>Δ3G>Δ5G. We detected no NAb response in two animals (Mm0301 in the Δ5G group and Mm0304 in the Δ3G group), and delayed and relatively weak responses in three animals (Mm0409 in the Δ5G group, Mm0511 in the Δ5G-ver1 group, and Mm0516 in the Δ3G group) (Fig. 2 B). Regardless of the levels of NAb, all 12 animals infected with the deglycosylation mutant viruses contained primary infection with similar kinetics (Fig. 2 A) suggesting that NAb were most likely not a critical factor for containment of the acute infection in these animals.

We previously found that animals vaccinated with Δ5G completely resist infection when challenged with the parental pathogenic SIVmac239 [3], showing minimal if any replication of the challenge virus for more than 10 years. A similar homologous challenge was performed in a subset of animals that received the deglycosylation mutants in the present study. Thus, one of the three “immunized” animals from each group was challenged with a high dose (1000 TCID_50_) SIVmac239 at 40 weeks following “vaccination” and plasma viral loads were determined (Fig. 3). As previously reported with Δ5G, a near-sterile immunity against challenge with SIVmac239 was not only noted with the Δ5G but also seen with our other three new deglycosylated SIV mutants, Δ5G-ver1, Δ5G-ver2 and Δ3G (Fig. 3). These results indicate that all 3 new vaccine versions possess similar equally high protective potential against the homologous, wild type SIVmac239 as the original Δ5G.

### Protection of the vaccinated macaques against heterologous challenge infection

**Figure 4:**
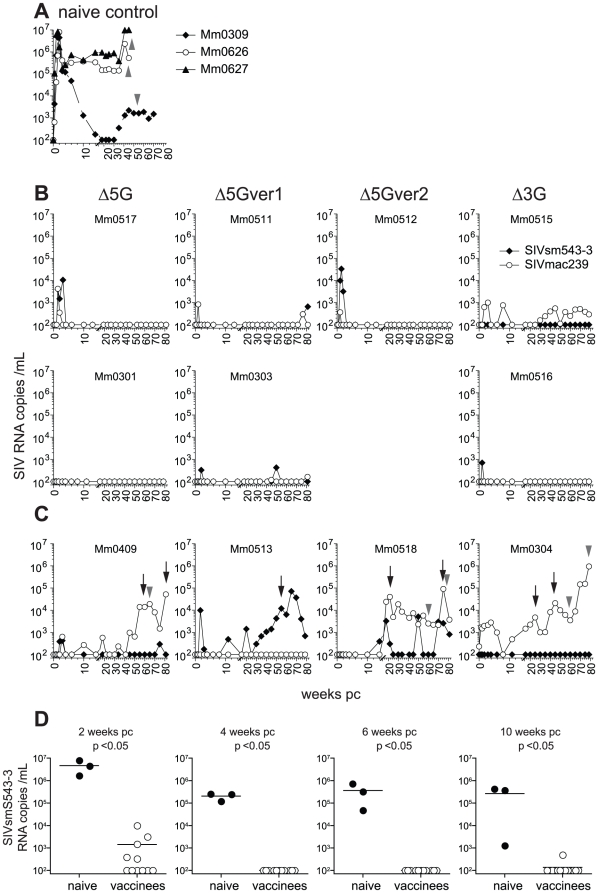
Plasma viral RNA loads in the heterologous SIVsmE543-3 challenge. Three vaccine-naïve animals (Mm0309, Mm0626, Mm0627) (A) and 11 vaccinees (B, C) were challenged intravenously with 1000 TCID_50_ of SIVsmE543-3. The vaccinees were divided into controllers (B) and non-controllers (C) based on control of vaccine and heterologous challenge infection. VL were determined by two sets of real-time RT-PCR for gag sequence of SIVsmE543-3 (closed diamonds) and SIVmac239 (open circles) respectively. For analysis of SIV sequence, PBMC and plasma were collected at the time-points indicated by arrows and arrowheads, respectively. (D) VL of the 11 vaccinees were statistically compared with those of 3 vaccine-naïve controls at 2, 4, 6 and 10 weeks pc. Significant difference between vaccinees and controls at each time points are shown (Mann-Whitney test).

The remaining eight animals (2 per group) vaccinated with each of the 4 vaccine versions (Δ5G, Δ5G-ver1, Δ5G-ver2 or Δ3G) and 3 of the four animals that were vaccinated (described in the above paragraph) and challenged with SIVmac239 (Mm0307 died of SIV unrelated causes) were challenged with a high dose (1000 TCID_50_) of SIVsmE543-3 delivered intravenously. Additional three naïve animals served as a control for this heterologous challenge experiment (Fig. 4 A). VL were monitored until 80 weeks post challenge (pc) using real time RT-PCR primer pairs and probes that distinguished the detection of SIVmac239 and SIVsmE543-3.

The 3 naïve control macaques infected with SIVsmE543-3 exhibited a peak VL of ∼10^7^ copies/ml at 2 weeks pi which is essentially similar to those we have routinely noted following infection with SIVmac239 with a few exceptions. Notably, the set point VL in SIVsmE543-3 was more than 10^5^ copies/ml in 2 animals which is at least 1-log higher than that noted in animals infected with SIVmac239 (Figs. 3 and 4 A). We reason that SIVsmE543-3 is likely to be more pathogenic than SIVmac239 for our Burmese rhesus macaques. Indeed, these 2 animals developed AIDS and were euthanized at 46 weeks pi, which is significantly faster than the time that we have noted for disease progression following SIVmac239-infection of these Burmese monkeys [3], [8], [15], [16], [17].

All 11 vaccinated animals contained the primary challenge with SIVsmE543-3 (Figs. 4A, B, and C). At 2 weeks pc, average VL for the vaccine groups were >3-log lower than those of naïve control animals (Fig. 4D). Thus, 5 of the 11 vaccinated animals (Mm0511, Mm0515, Mm0301, Mm0518, and Mm0304) contained the acute challenge below the level of detection (100 copies/ml) at all times, 3 animals (Mm0303, Mm0516, and Mm0409) showed marginal replication of SIVsm543-3 (329–700 copies/ml) during weeks 1–3 pc, while the other 3 animals (Mm0517, Mm0512, and Mm0513) showed viremia with 1–3×10^4^ copies/ml during weeks 1–3 pc but these replication peaks were transient and by 4 weeks pc, all vaccinated animals controlled SIVsmE543-3 to undetectable levels (Figs. 4B, C and D). Interestingly, transient replication of the vaccine viruses was also detected in 6 animals after the SIVsmE543-3 challenge (Mm0517, Mm0511, Mm0512, Mm0515, Mm0409, and Mm0304) (Figs. 4 B and C), suggesting reactivation of the deglycoylated virus upon super-infection with SIVsmE543-3. Statistical analysis of these data led us to conclude that the vaccinated animals significantly controlled SIVsmE543-3 replication at least during the acute phase up to 10 weeks pc irrespective of their MHC genotypes (Fig. 4 D). These results, taken together indicate that each of the deglycosylated vaccines utilized has the potential of inducing protective immunity against a potentially highly pathogenic heterologous challenge virus.

We next evaluated the containment of the challenge virus infection during the chronic-phase compared with the one observed after homologous challenge with SIVmac239 (Fig. 3 and ref [3]). Based on longitudinal VL (of either vaccine or challenge virus), the vaccinated animals were divided into two groups. One group of 7 animals, which we termed as “controllers”, comprised animals which controlled the challenge virus almost completely for the 80 weeks of follow-up pc (Fig. 4 B). Detailed analyses of VL showed complete control of the SIVsmE543-3 challenge in two of the animals (Mm0301 and Mm0515) over time. Similar potent antiviral control (except for small VL peaks during the acute-phase) were noted in three of the animals (Mm0512, Mm0516 and Mm0517). The last two “controllers” showed only occasional VL blips during the chronic-phase (Mm0303 and Mm0511). However, challenge with SIVsmE543-3 induced persistent low vaccine VL in Mm0515, while the challenge virus remained undetectable.

In what we termed as the non-controller group of 4 animals, VL gradually increased with time (Fig. 4 C). The evolving replicating viruses were found to consist of the challenge virus in Mm0513, whereas they were apparently vaccine viruses in the remaining three (Mm0409, Mm0518 and Mm0304). In the latter three, it appeared as if vaccine viruses were reactivated upon challenge with heterologous virus. These three eventually developed AIDS and were euthanized, whereas Mm0513 has not shown any disease manifestation >80 weeks pc. These four animals were regarded as poor or non-controllers.

These results indicate that the deglycosylated, live attenuated SIV viruses function as effective vaccines and possess potential to induce near-sterile, long-lasting immunity against the heterologous virus in a significant, albeit not all vaccinated animals. Also these results demonstrated that all 4 deglycosylation mutants exhibited similar vaccine efficacy based on the ratio of controllers and non-controllers (Figs. 4 B and C).

### Adaptive immune responses in vaccinees

**Figure 5:**
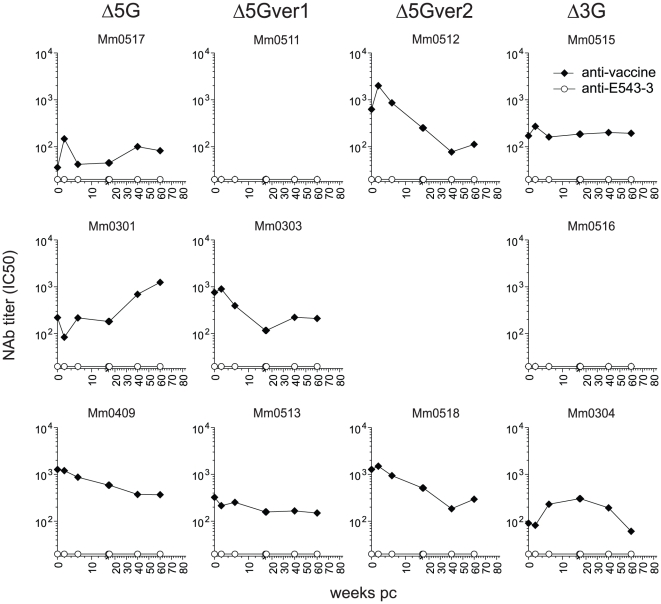
Neutralizing antibodies (NAb) response in the vaccine recipients. NAb responses against vaccine viruses (closed squares) and challenge virus (open circles) were measured in CEMx174/SIVLTR-SEAP system. NAb titers were indicated as the reciprocal of the dilutions of the plasma from the vaccinees yielding 50% inhibition (IC_50_).

In efforts to investigate immune correlates of protection against the heterologous challenge in vaccinees during acute and chronic infection, we examined adaptive immune responses against vaccine and challenge viruses. As described, the levels of NAb responses against vaccine virus varied among the vaccinees, partly due to the differences in N-glycosylation in gp120 (Figs. 1 and 2B). The differences in the NAb responses in vaccinees were maintained even after challenge with the heterologous virus. Whereas the Δ5G-ver2-vaccinated animals elicited the highest level of NAb, the Δ3G-vaccinated animals elicited the lowest level of NAb. In addition, the Δ5G and Δ5Gver1-vaccinated animals elicited intermediate NAb responses (Fig. 5). Regardless of these differences, all of the vaccinees successfully contained acute-phase VL, before diverging into controllers and non-controllers during the chronic infection-phase (Figs. 4 B and C). Thus, vaccine induced NAb responses did not correlate with protection from challenge virus infection during either acute or chronic infection. In addition, we could not detect any appreciable NAb against SIVsmE543-3 in any of the vaccinees throughout the observation period (Fig. 5). Although NAb was reasoned to exert immune pressure driving the emergence of mutants with altered N-glycosylation in HIV/SIV infections [18], [19], [20], no significant association was observed between NAb responses and the emergence of the mutants in non-controllers (Figs. 4 and 5).

**Figure 6:**
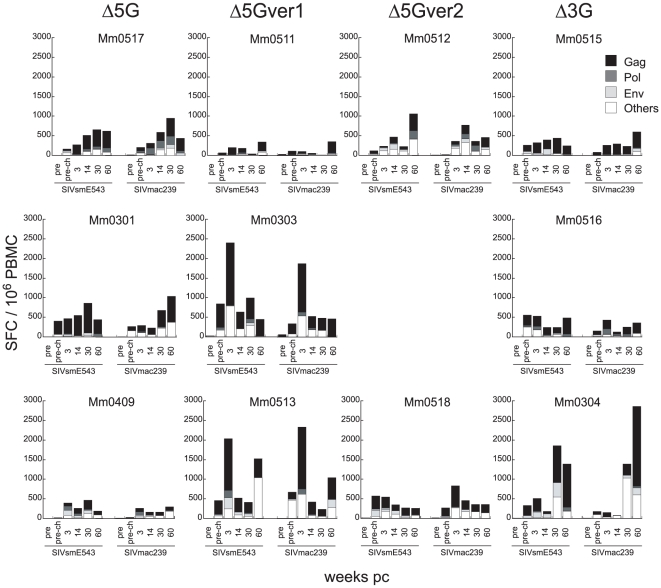
SIV specific cellular response in the vaccine recipients. SIV specific T cells were stimulated with overlapping peptides encompassing the viral proteins (Gag, Pol, Env, and Others: Vif, Vpr, Vpx, Tat, Rev, and Nef) of SIVmac239 and SIVsmE543-3 and the number of spot forming cells (SFC) per 10^6^ PBMC determined utilizing the IFN-γ ELISPOT assay. The PBMC samples analyzed for the responses included those collected pre-vaccination, pre-challenge (4–8 weeks prior to the challenge) and at 3, 14, 30 and 60 weeks pc.

We next examined cellular responses specific to the viral proteins in the PBMC utilizing the IFN-γ ELISPOT assay against pools of peptides spanning the entire proteins of both SIVsmE543-3 and SIVmac239. Specific T cell responses to SIVmac239-peptides paralleled those to SIVsmE543-3-peptides in all of vaccinees, and therefore vaccine-elicited SIV specific T cells were assumed to cross-react with SIVsmE543-3 infected cells (Fig. 6). However, no obvious quantitative correlation was found between the overall specific T cell responses and either good, poor or the lack of control of viremia throughout the observation period (Fig. 4). It is of interest to note that more than half of the SIV specific T cell responses appeared directed against epitopes localized within the SIV-Gag protein in most of the vaccinated animals (Fig. 6) which suggests that a potential association exists between gag specific T cell response with control of viremia. These findings are consistent with previous reports that suggest that the magnitude of Gag-specific T cell response correlates with control of HIV/SIV viremia in not only HIV-1-infected cohorts [11], [21] but also in macaques included in vaccine studies [8], [16].

Taken together, whereas these results indicate no appreciable correlation between NAb response and control of heterologous challenge intravenous infection, there may exist a potential role of virus specific cellular responses in the control of viral replication.

### Emergence of escape mutants with increased N-glycosylation sites in gp120 by recombination and single point mutations

**Figure 7:**
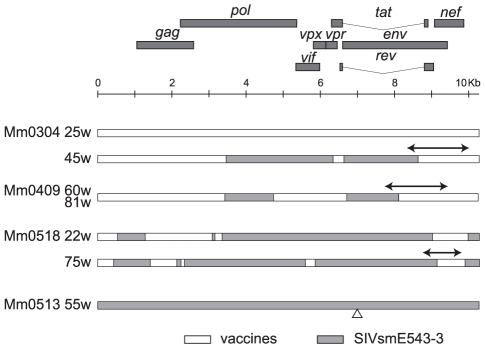
Recombination between vaccine and challenge virus. Nucleotide sequences of SIV fragments amplified by sets of nested PCR using primers based on SIVsmE543-3 or SIVmac239 to cover the entire SIV genome except the 5′ and 3′ terminal sequences (∼100 bp). Representative sequences from integrated results of multiple sequences of PCR fragments are shown. The sequences detected in Mm0304 at 25 weeks pc were the vaccine virus sequences (top bar indicated by open box) and the sequences detected in Mm0513 at 55 weeks pc were the challenge virus sequences (bottom bar indicated by grey box) except in the case of the latter which contained a 9 bp deletion shown by triangle. Other represented sequences were recombinant viruses between vaccine (open boxes) and SIVsmE543-3 (gray boxes). Lines with arrowheads indicate the sequences that were targeted for nested PCR to quantify the recombinant viruses and SIVsmE543-3 in the non-controllers.

In efforts to understand the mechanisms involved in the loss of control of viremia in the 4 non-controllers, we sequenced the emerging viruses using PBMC collected at time points indicated by arrows in Fig. 4 C. Sequence analysis of viruses isolated from Mm0513 confirmed that only the challenge virus with a 9 nucleotide deletion was replicating in this animal (Fig. 7). Whereas the vaccine virus was detected in the PBMC from Mm0304 collected at 25 weeks pc, a recombinant virus was predominantly present in the PBMC samples collected at 45 weeks pc from this animal (Fig. 7). Viruses with multiple recombinations were also found in Mm0409 and Mm0518 (Fig. 7). To examine if the recombination that we detected in the PBMC DNA was representative of the replicating viruses, we performed nested PCR utilizing primer pair sets aimed at the detection of putative recombination sites on RNA obtained from plasma from each animal (File S1). Consistent with the results obtained from PBMC DNA, the recombinants were also found in plasma RNA samples in all 3 animals, whereas only a few SIVsmE543-3 sequences were detected in Mm0518 (Table 2).

**Table 2:** SIVsmE543-3 derived and recombinant viruses in non-controllers^a^.

Vaccinee	Weeks pc	SIVsmE543-3	Recombinants
Mm0304	60	0	320
	80	0	1600
Mm0409	60	0	200
Mm0518	60	3	60
	75	3	200

a Viral RNA in plasma was converted to cDNA, serially diluted and subjected to nested PCR to quantify SIVsmE543-3 and the recombinant viruses between the vaccine and SIVsmE543-3. Frequencies of SIVs were estimated as the total viral sequences detected by nested PCR using cDNA synthesized from 0.128 ml of plasma.

**Figure 8:**
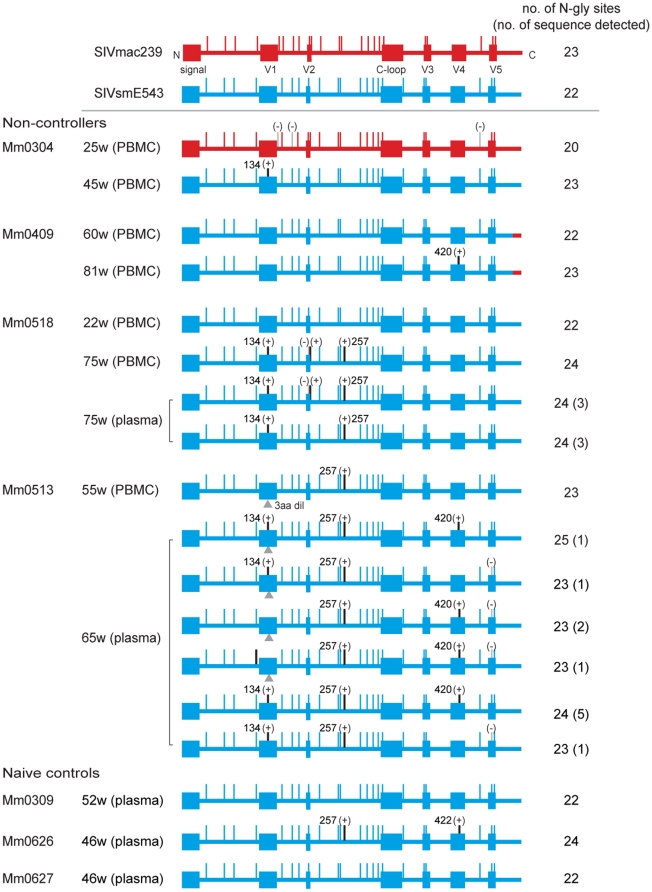
N-glycosylation sites in gp120 of the emerged viruses. The putative N-glycosylation sites (vertical bars) in gp120 were analyzed based on sequencing data from PBMC and plasma samples obtained from non-controllers. The red lines indicate SIVmac239 or vaccine virus sequences, and blue lines indicate SIVsm534-3 sequences. The notations (−) and (+) indicate the loss and addition of N-glycosylation site respectively. The triangle sign indicates a 3 amino acid deletion located within the V1 region found in the Mm0513. The numbers shown besides (+) denote the amino acid sequence numbers for hotspots of addition of N-glycosylation sites based on SIVsmE543-3 Env amino acid sequence (accession no. U72748). The total number of N-glycosylation sites found in each sequence was shown on the right, and the numbers in parenthesis indicate the number of sequences detected by PCR.

As noted above, while attenuated vaccine viruses have 18 or 20 N-glycosylation sites, the pathogenic strains, SIVmac239 and SIVsmE543-3 have 23 and 22 *N*-glycosylation sites, respectively (Fig. 1). We noticed that the gp120-encoding region of all replicating viruses in the chronic phase post challenge originated from the SIVsmE543-3 isolate regardless of recombination (Fig. 7). This resulted in restoration of N-glycosylation sites, the number of which was analyzed. We sequenced the PCR products amplified from plasma RNA shown in Table 2 and plasma RNA from naïve control animals (the time-points chosen for analysis were shown by arrowheads in Figs. 4A and C). The newly replicating viruses were now found to possess restored numbers (>22) of N-glycosylation sites (Fig. 8). Mutation associated with N-glycosylation in gp120 of SIVsmE543-3 was a common feature of the SIV that we detected in non-controllers regardless of the occurrence of the recombination events. Accordingly, the viruses detected at later time-points had increased number of N-glycosylation sites compared with those from earlier time-points. For virus sequences that included 23 to 25 sites, additional N-glycosylation sites were acquired by single point mutations (Fig. 8). Of note, the addition of N-glycosylation sites preferentially occurred in the following three regions: V1, between V2 and the C-loop and V4 (Fig. 8). We also found a viral sequence with 2 additional N-glycosylation sites that reside within these hotspots in one of the naïve control animals (Mm0626) (Fig. 8). These results indicate that mutations associated with glycosylation of gp120 were associated with persistent viral replication during the chronic phase in all of the non-controllers and one of three naïve controls. These data suggest that glycosylation plays a prominent role in optimizing fitness and/or evasion from vaccine-induced host responses in these viruses.

## Discussion

In this study, we examined whether reduction of glycosylation on viral spikes would allow for more ready access for host immune responses and thus provide for a new type of live attenuated vaccine, which would induce more robust anti-viral immune response and protect outbred rhesus macaques, against heterologous virus challenge. To evaluate the influence of allelic differences in host genetic properties on the efficacy of the vaccine, we used rhesus macaques with defined MHC-I and -II genes. Irrespective of differences in MHC genotypes, following a transient primary infection in approximately half of the vaccine recipients, all 11 vaccinated animals suppressed the acute-phase viral replication below the level of detection between 4 and 10 weeks pc (Figs. 4 B, C and D). After 10 weeks pc, containment of challenge virus infection diverged in two ways: 1) the majority (7 of the 11) of the vaccinated animals continued to control heterologous virus for more than 80 weeks pc without the contribution of elite MHC alleles previously associated with spontaneous CD8+ T cell mediated control of SIV replication in chronic SIV infection [6], [7], [22] (Fig. 4 B); 2) however, 4 of the vaccinated animals showed re-activation of SIV replication (Fig. 4 C) and three eventually developed AIDS. These results demonstrate that the host responses induced by these vaccines are capable of protecting from heterologous challenge virus at least during a limited period shortly after the challenge (10 weeks) irrespective of the inherited genetic properties of the host. However due to quantitative and/or qualitative changes in the protective response during the chronic-phase, viruses overcome the protective host response and most likely evolve viral diversity that is resistant to virus specific immune responses.

The pathogenic viral replication was associated with the emergence of viruses that were recombinants between the vaccine virus and the challenge virus SIVsmE543-3 and was associated with an increased glycosylation of viral spikes (Figs. 7 and 8). On the other hand, a significant number of the vaccinated animals controlled the infection with SIVsmE543-3 not only during the primary-phase but also during the chronic-phase (Fig. 4 B). We speculate that the properties of the recombinants might be essential for these viruses to circumvent the protective responses in these three vaccinees, which were able to control both the vaccine and the challenge viruses. Interestingly, the recombinant viruses shared the common part of Pol and Env from SIVsmE543-3 and that of Gag and Nef from the vaccine viruses (Fig. 7). The complexity of the recombination patterns between vaccine and challenge viruses suggest that repeated events of super-infection with both viruses replicating concurrently must occur to allow for recombination to occur. Subsequently, only a few viruses that succeeded in evading vaccine-induced host responses have managed to replicate to substantial levels in these non-controllers. These viruses are assumed to have acquired distinctive properties conferred by the chimera structures that make them different from the original vaccine and challenge viruses. Viral spikes derived from SIVsmE543-3 with increased glycosylation are likely essential for these properties, since all of the SIV sequences examined at later time points had more than 23 N-glycosylation sites in gp120 of SIVsmE543-3 (Fig. 8). Although the changes of N-glycosylation sites in gp120 of SIV/HIV have been previously reported to be associated with escape from neutralizing antibody response [7], [19], [20], we did not detect any significant titers of neutralizing antibodies against the challenge virus in any of the 11 vaccinees (Fig. 5). Thus, modification of N-glycosylation sites in these viruses might play a role in inducing anti-viral host responses other than neutralizing antibody responses, such as antibody-dependent cell-mediated cytotoxicity (ADCC) or such modifications might lead to altered viral properties or “viral fitness” such as tissue/cell tropism, replication levels, and/or stability in these animals.

It remains to be elucidated why an established immunity capable of containing the viral burst during acute infection gradually loses its grip over the virus, allowing for the generation of these mutant viruses. Select genetic properties required for prevention of emergence of escape mutants and/or viruses with altered fitness able to overcome vaccine-induced protective host responses might be lacking in these four animals. Indeed, MHC I allele A1*0560202 and the ones associated with MHC II haplotype 89002-p such as A1*01807 and others were identified only in non-controllers (File S1). Similarly, MHC I alleles such as A1*0040102, A1*11001, A1*03202 were indentified in controllers but not non-controllers suggesting immune responses regulated by MHC genes such as CTL and NK via KIR related mechanisms might play a role in viral control during the chronic-phase. Our attempts to identify immunological correlates of protection suggested that magnitude of overall T cell responses could not account for either the marked containment of the infection during the acute-phase or the different outcome of the infection between controllers and non-controllers during the chronic-phase. Nevertheless, the fact that Gag-specific T cells constitutes more than 50% of the repertoire of SIV specific T cells in the vaccinees (Fig. 6) suggest an important role for these responses in the containment of the heterologous virus infection. The magnitude of such responses may also be critical during acute infection illustrated by the association of strong acute cellular response and rare detection of recombinant virus in one vaccinee Mm0513, that prevented disease progression (Figs. 4 C and 6).

Efficacy of a live attenuated vaccine against heterologous virus has also been studied using nef gene deleted mutants including SIVmac239Δnef [23] and SIVmac239Δ3 [24] as live attenuated vaccines. It is difficult to compare those results with results of the studies reported herein obtained under similar but not exactly the same conditions. However, a number of differences between the use of SIVΔnef and the studies reported herein were noted: First, the control of acute-phase viral infection occurred in the vaccinees with MHC I alleles associated with elite controller, Mamu B*08 and B*17 in the Δnef vaccine study. In contrast, our study indicated control of the primary infection in all of the vaccinees irrespective of the diversity of MHC genotypes. Second, the containment of the challenge virus in the chronic-phase also appeared to be much less prominent in these two studies. These differences might stem from the intrinsic properties of the two types of live attenuated SIV. We have previously reported that SIVmac239Δnef and SIVmac239 with a functional *nef* gene replicate preferentially in B cell areas and T cell areas, respectively, in peripheral lymph nodes during primary infection [17]. On the other hand, Δ5G replicates preferentially in CD4+T cells in intestinal effector sites such as lamina propria, whereas the wild-type SIVmac239 replicates in CD4+T cells in inductive sites such as T cell areas of secondary lymphatic tissues. These subtle differences of tissue and cell tropism suggests that the mechanisms of attenuation may differ between Δ5G and SIVmac239Δnef and may further explain why the latter is likely more pathogenic than the former. In addition, the differences in the susceptibility of the macaques to SIV, estimated by the magnitude of peak VL during primary infection and set point VL, could be another factor that influenced the results of the studies. In nonhuman primate model for AIDS, the properties of SIV strains and the origin of macaques appear to affect the results and interpretation of the data from the experiments. Judged from previous studies from a number of other laboratories including ours that have utilized Burmese [8], [16], [17] and Indian macaques [6], [25], [26], [27] respectively, Burmese rhesus macaques infected with SIVmac239 tend to have lower set point VL and require more time to develop AIDS than Indian rhesus macaques. Thus, these differences might have allowed us to discover potent protective host responses against heterologous virus elicited by a deglycosylated live-attenuated vaccine. On the other hand, this study also demonstrates that Burmese macaques were more susceptible to SIVsmE543-3 than SIVmac239 (Figs. 3 and 4 A). In fact, these results indicate that SIVsmE543-3 and SIVmac239 might form an excellent model of heterologous challenge virus and a template virus to create vaccine viruses. These results also underline that macaque susceptibility to SIV might be more SIV strain specific than previously considered.

In summary, we report here for the first time, the induction of potent protective immunity against heterologous challenge by live attenuated SIV in macaques with a diverse MHC genetic background. Our system provides a unique and robust experimental paradigm for defining the potential immunological correlates of protection, assessing cross-subtype protection and designing HIV vaccines. However, emergence of pathogenic revertants from live attenuated SIVs by spontaneous mutations as well as by recombination has often been encountered in macaque AIDS models [23] [28] and certainly during our study. Thus, while a live vaccine strategy is clearly not a viable approach to actual HIV vaccine development, much can be learnt with regards to the mechanisms involved. As noted above, continuous stimulation of the host immune system by persistently infected vaccine virus at low levels may be a key factor for maintaining protective immunity not only against homologous but also heterologous SIV over a long period. We believe that creating such a condition, for instance, by a virus vector capable of establishing a persistent infection may be one strategy that may lead to the development of an effective vaccine against HIV. Minimally, the heterologous virus challenge model described herein provides a powerful tool to attempt to identify the potential mechanisms that lead to protective versus non-protective immunity. We reason that such events are likely to have occurred during the acute phase of “vaccine” virus replication which sets the course for the eventual response of the animals to the heterologous challenge virus. A detailed study of events that transpire during the acute infection period may provide unique insights on this issue.

## Materials and Methods

### Mean distance of amino acid sequences of HIV-1 group M subtypes and amino acid differences between SIVmac239 and SIVsmE543-3

The complete genome sequence alignments consist of 368 HIV-1 isolates (59 subtype A, 71 subtype B, 148 subtype C, 39 subtype D, 6 subtype F1, 3 subtype F2, 6 subtype G, 3 subtype H, 2 subtype J, 2 subtype K, 15 CRF01_AE, and 14 CRF02_AG) as determined from HIV sequence database (http://www.hiv.lanl.gov/cgi-bin/NEWALIGN/align.cgi) were used for these analyses. The alignment data was coordinated with HXB2-LAI-IIIB. These data led to the identification of nine coding regions, as determined utilizing the MEGA4 software [**29**]. We estimated the number of amino acid differences per site from averaging the over all sequence pairs between and within each subtype or CRF, and also mean diversity. All results are based on the pairwise analysis of the sequences, and standard error estimates were obtained by a bootstrap procedure (500 replicates). All positions containing gaps and missing data were eliminated from the dataset. The amino acid comparisons in each viral protein between SIVmac239 (Genbank accession no. M33262) and SIVsmE543-3 (Genbank accession no. U72748) were analyzed by Clustal W (http://www.clustal.org).

### Attenuated vaccine viruses and challenge virus

The molecular pathogenic clone of SIVmac239 [30] and its derived deglycosylated mutants used in this study are depicted in Fig. 1. The Δ5G was derived by site-directed mutagenesis of an SIVmac239 infectious DNA clone so that the asparagine residues for the N-glycosylation sites at aa 79, 146, 171, 460 and 479 in gp120 were converted to glutamine residues [2], [3]. The Δ5G-ver1, Δ5G-ver2 and Δ3G were also constructed by site-directed mutagenesis from the series of deglycosylated mutants reported previously [2]. The stocks of deglycosylated mutants were prepared by DNA transfection of respective proviral DNAs into 293T cells. The stock of SIVsmE543-3 was prepared as previously described [4]. These virus stocks were propagated in phytohemagglutinin-stimulated peripheral PBMC from rhesus macaques as previously described [3], [16].

### Animals

Juvenile rhesus macaques originating from Burma were used following negative results of screening for SIV, simian T-cell lymphotropic virus, B virus, and type D retrovirus infection prior to study inception. All animals were housed in individual cages and maintained according to the rules and guidelines for experimental animal welfare as outlined by National Institute of Infectious Diseases and National Institute of Biomedical Innovation, Japan. Full details of the study were approved (Approval number: 507006) by National Institute of Infectious Diseases Institutional Animal Care and Use Committee in accordance with the recommendations of the Weatherall report. Early endpoints are adopted including frequent monitoring of viral loads and immunological parameters, and humane euthanasia is conducted once any manifestation of clinical AIDS or signs of fatal disease is noted.

### Vaccination and challenge infection

Three animals per group were intravenously inoculated with 100 TCID_50_ of either of 4 deglycosylation mutants (Δ5G, Δ5G-ver1, Δ5G-ver2 and Δ3G) as shown in Fig. 1. At 40 weeks post infection, 4 SIV-infected animals: Mm0301 (Δ5G), Mm0513 (Δ5G-ver1), Mm0307 (Δ5G-ver2), Mm0304 (Δ3G) and three naive animals (Mm0608, Mm0521, and Mm0522) were intravenously inoculated with 1000 TCID_50_ of SIVmac239 for purposes of homologous virus challenge studies.

To examine the efficacy of the live attenuated vaccine against heterologous virus, 11 vaccinees were intravenously inoculated with 1000 TCID_50_ of SIVsmE543-3.3 as follows: Mm0517 (Δ5G), Mm0511 (Δ5G-ver1), and Mm0512 (Δ5G-ver2) were challenged at 50 weeks post vaccination with the deglycosylation mutant; Mm0409 (Δ5G), Mm0303 (Δ5G-ver1), and Mm0518 (Δ5G-ver2) were challenged at 61 weeks post vaccination; Mm0515 (Δ3G) and Mm0516 (Δ3G) were challenged at 117 weeks post vaccination. 3 naïve animals (Mm0309, Mm0626, Mm0627) were infected with SIVsmE543-3 as vaccine-naïve controls. Furthermore, three of SIVmac239-challenged animals, Mm0301, Mm0513 and Mm0304 (Mm0307 died with a SIV-infection-unrelated cause) were re-challenged with SIVsmE543-3 at 117 weeks post vaccination and 77 weeks post SIVmac239 challenge.

### Plasma viral load measurements

SIV infection was monitored by measuring the plasma viral RNA load using a highly sensitive quantitative real-time RT-PCR. Viral RNA was isolated from plasma samples from infected animals using MagNA PureCompact Nucleic Acid Isolation Kit (Roche Diagnostics). Real-time RT-PCR was performed by using QuantiTec Probe RT-PCR kit (Qiagen) and Sequence detection system SDS7000 (Applied Biosystems). To detect SIVmac239 gag and SIVsmE543-3 gag separately, primers and probe sets were synthesized as follow; SIVsmE543-3 gag specific primers: 5′- FAM-GCAGAGGAGGAAATTACCCAGTGC-3′, 5′-CAATTTTACCCAAGCATTTAATGTT- TAMRA- 3′ and probe 5′-TGTCCACCTACCCTTAAGTCCAA-3′, SIVmac239 specific gag primers: 5′-GCAGAGGAGGAAATTACCCAGTAC-3′, 5′-CAATTTTACCCAGGCATTTAATGTT-3′ and probe 5′-FAM-TGTCCACCTGCCATTAAGTCCCGA-TAMRA-3′. These primers and probes do not cross-react with SIVmac239 RNA and SIVsmE543-3 RNA. The detection sensitivity of plasma viral RNA by this method was calculated to be 100 viral RNA copies per ml of plasma.

### Sequencing of SIV RNA and proviral DNA

Viral RNA was isolated using MagNA PureCompact Nucleic Acid Isolation Kit (Roche Diagnostics) and cDNA was synthesized with two-step qRT-PCR kit (Invitrogen). PBMC from vaccine recipients were suspended with lysis buffer (10mM Tris 0.5% NP-40 and 0.5% Tween20) with Proteinase K (200 mg/ml), and incubated at 55°C for 1 hour, then heat-inactivated at 95°C for 5 min. Serial 10-fold diluted cDNA or cell lysate was subjected to nested PCR with the Ex-Taq PCR kit (Takara, Tokyo, Japan) with the following condition: 1 cycle of 97°C for 1 min. and then 25 cycles of amplification (94°C for 30 s, 55°C for 30 s, 72°C for 2.5 min) and 72°C for 10 min. and then 4°C for 5 min. Primers were designed to target the several overlapping sequences spanning the open reading frames of SIVmac239 or SIVsmE543-3 as shown in File S1. Positive PCR products were sequenced by using BigDye terminator cycle sequencing kits (Applied Biosystems) and analyzed by using ABI3100 or ABI 3130xl Genetic Analyzer (Applied Biosystems). Sequences were assembled using ATGC version 4.2 (Genetyx Corporation).

### SIV specific T cell responses

The T cells in the animals were examined for virus specific cellular response against the vaccine virus and the challenge virus by using pooled peptides covering overlapping sequences of all viral proteins of SIVmac239 and SIVsmE543-3 respectively. Briefly, cryopreserved PBMC were thawed, resuspended at 2×10^6^ cells/ml in R10 (RPMI1640 supplemented with 10% heat-inactivated FCS, 55 µM 2-mercaptoethanol, 50 U/ml penicillin and 50 µg/ml streptomycin), and rested for 2 h at 37°C. The cells were washed and aliquots of 10^5^ cells were stimulated with each pool of peptides at a final concentration of 2 mg/ml in an anti-IFN-g Ab-coated plate overnight. ELISPOT assay for the detection of IFN-g secreting cells were performed using a commercial ELISPOT kit (U-CyTech Bioscience). Peptides based on sequences of SIVmac239 viral proteins were synthesized by the Microchemical Facility, Emory University School of Medicine, Atlanta, GA, USA. Peptides based on the sequences of SIVsmE543-3 viral proteins were synthesized by Sigma-Aldrich Japan.

### Neutralization assay

Virus neutralizing antibodies were tested according to a protocol using CEMx174/SIVLTR-SEAP cells [31] as described previously [3]. Serially diluted heat-inactivated plasma was tested for inhibition of the corresponding vaccine virus or the challenge virus (SIVsmE543-3) in CEMx174/SIVLTR-SEAP cells. SEAP activity in the culture supernatant was assayed using a commercial SEAP reporter gene assay chemiluminescent kit (Roche Diagnostics).

### Statistical analysis

Correlation analysis was performed using Spearman's non-parametric rank test and Mann-Whiney ‘U’ test by using Graph pad Prism 4.0 software. Correlations were considered statistically significant when *P* values were <0.05.

### DNA sequence data deposition

The SIV sequences reported in this paper have been deposited in the DNA Data Bank of Japan (accession nos. AB553915 to AB554013).

## Supporting Information

File S1 (0.28 MB PDF)
