# Supplementary material for: Protection of Macaques with Diverse MHC Genotypes against a Heterologous SIV by Vaccination with a Deglycosylated Live-Attenuated SIV
Source: PLoS One. 2010 Jul 20;5(7):e11678. doi: 10.1371/journal.pone.0011678 (PMC2907403; doi:10.1371/journal.pone.0011678)
Supplement: File S1 — (0.28 MB PDF) [file pone.0011678.s001.pdf]

Supporting Materials and Methods

Analysis of MHC class I genes.

Total RNA was extracted from B-LCL prepared from each monkey and cDNA was synthesized by using SuperScript II reverse transcriptase (Roche). To investigate the Mamu-A and Mamu-B haplotypes, PCR products from Mamu-A or Mamu-B genes were analyzed by reference strand conformation analysis as described previously [1]. Briefly, the test cDNA fragment of 725 bp encoding extracellular domains of the MHC class I molecule was prepared by two-step PCR. The PCR products were then hybridized with two different fluorescence (Cy5)-labeled references prepared from the cDNA clone of Mamu-B*66 (FLR#1, GenBank Accession #AJ844597) and from the cDNA clone of Mamu-B*39 (FLR#2, GenBank Accession #AJ556890) and the heteroduplex DNAs were subjected to 6% Long Ranger gel (Cambrex BioScience Inc.) in 1xTBE (89 mM Tris-boric acid, 2 mM EDTA-Na_2, pH 9.0) mounted on ALF express II automated sequencing apparatus (Amersham Biosciences). For identification of Mamu-A and Mamu-B alleles, full-length cDNAs of Mamu-A and Mamu-B genes were amplified by PCR using locus-specific primer pairs, as described previously [1] with some modifications of primers as reported by Karl et al. [2]. In brief, for the Mamu-A gene we used 5'MHC_UTR (5'-GGACTCAGAATCTCCCCAGACGCCGAG) and 3'MHC_UTR_A (5'-CAGGAACAYAGACACATTCAGG), while 5'MHC_UTR and 3'MHC_UTR_B (5'-GTCTCTCCACCTCCTCAC) were used for the Mamu-B gene. The PCR products were cloned into pSTBlue-1 Perfectly Blunt vector (Novagen) according to the manufacturer's instructions. At least thirty independent clones for Mamu-A and Mamu-B genes were sequenced on both strands. When at least three clones showed identical sequences, we submitted the sequences to DNA Data Bank of Japan (DDBJ) data base (AB477383, AB477386, and AB477388 to AB477400) and to the Immuno-Polymorphism Database (IPD) for non-human primate MHC (http://www.ebi.ac.uk/ipd/mhc/submit.html) to obtain official nomenclature of Mamu-A and Mamu-B alleles.

Genotyping and haplotype analyses of MHC class II (Mamu-DRB, DQ, and DP) loci.

MHC class II alleles and haplotype compositions of the macaques used were analyzed by sequencing of cloned cDNA. Total RNA was extracted from B-LCL and cDNA was synthesized by using SuperScript II reverse transcriptase (Invitrogen). DRB, DQA, DQB, DPA, and DPB exon 2 fragments were amplified by PCR using KOD-FX (Toyobo) and the following primer sets designed to hybridize with the conserved monomorphic regions:

CACTGGCTKTGGCTGGGGACAC and GCAGGATACACAGTCACCTTAG for DRB, AGGTGAAGACATWGTSGCTGACCA and ACTTGGAAAACACTGTGACCT for DQA, CAACYGTGACCTTGATGCTGGC and AGGTTGTGGTGGTTGAGGGCCT for DQB, CTGCTGAGTCTCCGAGGAG and ACAGGCTCCTTGGGAAA for DPA, and GCCCGTCGACTCCATGATGGTTCT and AGGTTGTGGTGCTGTAAG for DPB. The PCR products were subcloned into pBluescript II SK(+) (Stratagene) and sequenced. When more than four clones with an identical sequence were obtained for a single allele, the allele was considered to be expressed in the animal. Nucleotide sequences of newly identified alleles have been deposited to the DDBJ database (AB544420, AB548912, AB548915, AB548927, AB548929, AB548931, AB548934, AB549238, and AB551650 to AB551652). Haplotype compositions of the MHC class II loci were estimated by comparing expressed alleles between parents and children in each ancestry tree.

References

1. Tanaka-Takahashi Y, Yasunami M, Naruse T, Hinohara K, Matano T, et al. (2007) Reference strand-mediated conformation analysis-based typing of multiple alleles in the rhesus macaque MHC class I Mamu-A and Mamu-B loci. Electrophoresis 28: 918-924.

2. Karl JA, Wiseman RW, Campbell KJ, Blasky AJ, Hughes AL, et al. (2008) Identification of MHC class I sequences in Chinese-origin rhesus macaques. Immunogenetics 60: 37-46.

Table S1. MHC-I genes

Vaccine	Δ5G			Δ5Gver1		
Animal#	Mm0301	Mm0409	Mm0517	Mm0513	Mm0511	Mm0303
A allele	A1*0040102<br>A1*11001	A1*10701<br>A1*0560202	A1*03202<br>A1*04904<br>A1*11001	A1*01807<br>A1*02603<br>A2*0103<br>A4*1403	A1*10502<br>A2*0546	A1*0040102<br>A1*01907<br>A2*0526
B allele	B*4301<br>B*9201N<br>B*38N<br>B*66N1<br>B*7301	B*1601N<br>B*4615N1<br>B*02N<br>B*6001N<br>B*0201	B*3301N<br>B*0401<br>B*6601<br>B*3901N	B*0101<br>B*0702	B*6601<br>B*1601N<br>B*4615N1	B*4301<br>B*6601<br>B*9201N<br>B*2602

Vaccine	Δ5Gver2			Δ3G		
Animal#	Mm0307[a]	Mm0512	Mm0518	Mm0304	Mm0515	Mm0516
A allele	A1*02806<br>A1*10502<br>A2*0511	A1*0040102<br>A1*05602	A1*01807<br>A1*00802<br>A2*0103<br>A4*1403	A1*0560202<br>A2*0503	A1*0040102<br>A1*05001<br>A2*0511<br>A4*1403	A1*03202<br>A1*11001
B allele	B*9601N<br>B*6601	B*8201N<br>B*4615N2<br>B*0201	B*0101<br>B*0702	B*7702	B*4301<br>B*9201N<br>B*0703<br>B*3901N	B*3301N<br>B*0401<br>B*38N<br>B*66N1

Challenge virus	SIVmac239			SIVsmE543-3		
Animal#	Mm0608	Mm0521	Mm0522	Mm0309	Mm0626	Mm0627
A allele	A1*10504<br>A1*0560202<br>A1*10502<br>A1*10503	A1*11201<br>A1*01807<br>A1*04301<br>A2*0103	A1*0080102<br>A1*0080103<br>A1*10701<br>A1*11301	A1*11101<br>A1*01808	A1*02203<br>A1*02603<br>A2*051504	A1*01808<br>A1*03203<br>A1*10901
B allele	B*0201<br>B*6601	B*0702	B*0702<br>B*3901	B*3701<br>B*4301<br>B*3602N<br>B*4503N<br>B*5102N<br>B*9201N<br>B*3901N<br>B*2602	B*0702<br>B*010102<br>B*1703	B*1301<br>B*6601<br>B*7002<br>B*8201N<br>B*8901

[a] Died before heterologous challenge by the cause unrelated to SIV infection.

Table S2. MHC-II genes and haplotypes

Vaccine	Δ5G					
Animal#	Mm0301		Mm0409		Mm0517	
Haplotype	90030-h	93006F1-2	89002-q	89075-s2	NN [a]	90010-d1
DRB	DRB1*0323<br>DRB1*0321	DRB1*0306<br>DRB*W2603<br>DRB1*07033	DRB5*0305<br>R170DR08	DRB*W3602<br>DRB3*0410	DRB1*0407<br>DRB1*0409	DRB*W2104<br>DRB*W2603<br>DRB*W606
DQA	DQA1*0104	DQA1*0105	DQA1*2404	DQA1*0108	DQA1*07	DQA1*0502
DQB	DQB1*0601	DQB1*0602	89002DQB-q	DQB1*0607	DQB1*1710	DQB1*1603
DPA	90030DPA02	9712DPA01	89002DPA01	9712DPA01	9712DPA01	0008DPA01
DPB	DPB1*12	DPB1*190102	DPB1*06	9703DPB01	DPB1*190102	90010DPBd1

Vaccine	Δ5Gver1					
Animal#	Mm0513		Mm0511		Mm0303	
Haplotype	89002-p	89018-1	90010-d2	89002-q	90030-h	90014-z
DRB	89002DRB-p1<br>DRB*W203	DRB1*0310<br>DRB*w602	DRB*W2104<br>DRB*W2604<br>DRB*W606	R170DR0301<br>R170DR08	DRB1*0323<br>DRB1*0321	DRB*W2505<br>DRB4*0104<br>DRB1*0704
DQA	89002DQA-p	DQA1*2302	DQA1*0502	DQA1*2404	DQA1*0104	DQA1*0503
DQB	DQB1*1812	DQB1*1804	DQB1*1503	89002DQB-q	DQB1*0601	DQB1*2401
DPA	DPA1*0601	DPA1*0701	90030DPA01	89002DPA01	90030DPA02	DPA1*0202
DPB	DPB1*04	DPB1*190102	DPB1*09	DPB1*06	DPB1*12	DPB1*06

Vaccine	Δ5Gver2					
Animal#	Mm0307[b]		Mm0512		Mm0518	
Haplotype	90010-d2	89075-s	89075-s2	90010-e2	89002-p	90069-1
DRB	DRB*W2104<br>DRB*W2603<br>DRB*W606	DRB*W101<br>DRB*W3602<br>DRB1*0320	DRB*W3602<br>DRB3*0410	DRB1*0309<br>DRB*W2507	89002DRB-p1<br>DRB*W203	DRB1*0306<br>DRB1*1003
DQA	DQA1*0502	DQA1*0108	DQA1*0108	DQA1*2601	89002DQA-p	DQA1*2602
DQB	DQB1*1503	DQB1*0607	DQB1*0607	DQB1*1801	DQB1*1812	DQB1*1811
DPA	90030DPA01	89075DPA01	9712DPA01	90010DPA-e	DPA1*0601	DPA1*0601
DPB	DPB1*09	89075DPB-s	9703DPB01	DPB1*18	DPB1*04	DPB1*04

Vaccine	Δ3G					
Animal#	Mm0304		Mm0515		Mm0516	
Haplotype	89075-t01	89075-s2	90030-h	90088-j	90010-d1	93006F1-2
DRB	DRB1*0320<br>DRB1*0320	DRB*W101<br>DRB*W3602<br>DRB3*0410	DRB1*0323<br>DRB1*0321	DRB1*0403<br>DRB1*W502	DRB*W2104<br>DRB*W2603<br>DRB*W606	DRB1*0306<br>DRB*W2603<br>DRB1*07033
DQA	DQA1*0108	DQA1*0108	DQA1*0104	DQA1*03	DQA1*0502	DQA1*0105
DQB		DQB1*0607	DQB1*0601	DQB1*1808	DQB1*1603	DQB1*0602
DPA	89075DPA01	9712DPA01	90030DPA02	DPA1*0201	0008DPA01	9712DPA01
DPB	9703DPB01	9703DPB01	DPB1*12	DPB1*10	90010DPBd1	DPB1*190102

Challenge virus	SIVmac239					
Animal#	Mm0608		Mm0521		Mm0522	
Haplotype	90010-d2	89-075s	89-002-p	90120-a	89-002-q	90088-j
DRB	DRB*W2104<br>DRB*W2604	DRB*W3602<br>DRB3*0410	89002DRB-p<br>DRB*W203	DRB1*1007<br>DRB1*0303	R170DR03<br>R170DR08	DRB1*0403<br>DRB*W502
DQA	DQA1*0502	DQA1*0108	89002DQA-p	DQA1*05	DQA1*2404	DQA1*03
DQB	DQB1*1503	DQB1*0607	DQB1*1812	DQB1*1801	89002DQB-q	DQB1*1808
DPA	90030DPA01	9712DPA01	DPA1*0601	90010DPA-e	89002DPA01	DPA1*0201
DPB	DPB1*09	9703DPB01	DPB1*04	DPB1*	DPB1*06	DPB1*10

Challenge virus	SIVsmE543-3					
Animal#	Mm0309		Mm0626		Mm0627	
Haplotype	89002-q	90120-b01	89075-s	89018-1	90088-j01	90048-2
DRB	DRB5*0305<br>R170DR08	DRB*W2002<br>DRB*W2508	DRB*W101<br>DRB*W3602<br>DRB3*0410	DRB*W101<br>DRB1*0310<br>DRB*W609	DRB1*0403<br>DRB*W501	DRB1*0318<br>DRB*W604<br>DRB*W603
DQA	DQA1*2404	DQA1*0502	DQA1*0108	DQA1*2302	DQA1*03	DQA1*2404
DQB	89002DQB-q	DQB1*1603	DQB1*0607	DQB1*1804	DQB1*1808	DQB1*1502
DPA	89002DPA01	9712DPA01	89075DPA01	DPA1*0701	DPA1*0201	DPA1*0401
DPB	DPB1*06	DPB1*190102	89075DPB-s	DPB1*190102	DPB1*10	DPB1*13

[a] Not named haplotype

[b] Died before heterologous challenge by the cause unrelated to SIV infection.

Table S3. MHC genes in controllers and non-controllers

Controller

Animal#		Mm0301	Mm0303	Mm0512	Mm0515	Mm0516	Mm0517	Mm0511
MHC II haplotype		90030-h<br>93006F1-2	90030-h<br>90014-z	89075-s<br>90010-e	90030-h<br>90088-j	90010-d1<br>93006F1-2	90010-d1<br>NN [a]	89-002-q<br>90010-d2
MHC I alleles	A	A1*0040102<br>A1*11001	A1*0040102<br>A1*01907<br>A2*0526	A1*0040102<br>A1*05602	A1*0040102<br>A1*05001<br>A2*0511<br>A4*1403	A1*03202<br>A1*11001	A1*03202<br>A1*04904<br>A1*11001	A1*10502<br>A2*0546
	B	B*4301<br>B*9201N<br>B*38N<br>B*66N1<br>B*7301	B*4301<br>B*6601<br>B*9201N<br>B*2602	B*8201N<br>B*4615N2<br>B*0201	B*4301<br>B*9201N<br>B*0703<br>B*3901N	B*3301N<br>B*0401<br>B*38N<br>B*66N1	B*3301N<br>B*0401<br>B*6601<br>B*3901N	B*6601<br>B*1601N<br>B*4615N1

[a] Not named haplotype

Non-controller

Animal#		Mm0409	Mm0304	Mm0518	Mm0513
MHC II haplotype		89002-q<br>89075-s	89075-t1<br>89075-s	89002-p<br>90069-1	89002-p<br>89018-1
MHC I alleles	A	A1*10701<br>A1*560202	A1*560202<br>A2*0503	A1*01807<br>A1*00802<br>A2*0103<br>A4*1403	A1*01807<br>A1*02603<br>A2*0103<br>A4*1403
	B	B*1601N<br>B*4615N1<br>B*02N<br>B*6001N<br>B*0201	B*7702	B*0101<br>B*0702	B*0101<br>B*0702

Table S4. Primers for nested PCR to amplify SIV sequences

SIVmac239

		Nucleotides	Primers	Sequence (5' -> 3')
1	1st	20-1519	F20	TGCAAGAAGACATAGAATCTTAGACA
			R1519	GCATTTAATGTTCTCGGGCTTAAT
	2nd	49-1381	F49	ACTTAGAAAAGGAAGAAGGCATCAT
			R1381	ACCACTAGGTGTCTCTGCACTATCT
2	1st	859-2235	F859	TCCTGAGTACGGCTGAGTGAAGGC
			R2235	TCCAACACTTAATTGGCTTTCTTG
	2nd	895-2189	F895	AGGAACCAACCACGACGGAG
			R2189	AAAGGGATTGGCACTGGTGCGAGG
3	1st	2057-3492	F2057	AAGGGGCTGGGTGTGAATCCCACC
			R3492	AACCCTATGCTATTCAAGAGTTCC
	2nd	2092-3453	F2092	TGCTGACGGCTTGTCAAGGAGTAGG
			R3453	ACCCTGTCATGTTCCAGGTCTGTCC
4	1st	3314-4775	F3314	GGGTCACCAGCCATCTTCCAATAC
			R4775	GAATCCACTAGCTACATGTACTGC
	2nd	3359-4727	F3359	CCCTTCAGGAAGGCAAATCCAGATG
			R4727	GGGTACAATCCATTTGCCAAGTCCC
5	1st	4591-5942	F4591	ATTGGTATTCAAATTTGGATTACC
			R5942	AGCTCCCTTGGTAGGTGGTTTACC
	2nd	4625-5883	F4625	GCCAGACAGATAGTAGACACCTGTG
			R5883	CCATTCGAAGGCCTCTCCTATTGTC
6	1st	5718-7126	F5718	AGGGGAGAACAACTGCTGTCAAGC
			R7126	ACCCTGTCATGTTGAATTTACAGC
	2nd	5751-7085	F5751	CCGAGAGCTCATAAGTACCAGGTAC
			R7085	TCCAAGCCTGTGCAATTATCCTGGG
7	1st	6721-8394	F6721	ATTCCCCTCTTTTGTGCAACCAAG
			R8394	CTCGATGGCAGTGACCCTAGTCTG
	2nd	6757-8356	F6757	TGGGGAACAACTCAGTGCCTACCAG
			R8356	CCCAGACGGTCAGTCGCAACAATTC
8	1st	8235-10004	F8235	CGCGGCGTCGTTGACGCTGACCGC
			R10004	CTGCCAGCCTCTCCGCAGAGCGAC
	2nd	8278-9947	F8278	GCTGGGATAGTGCAGCAACAGCAAC
			R9947	ATCAAGAAAGTGGGCGTTCCCGACC
9	1st	9322-10094	F9322	CAGAAAACAAAATATGGATGATATAGATG
			R10094	TAACAGACCAGGGTCTTCTTATTATTGAGT
	2nd	9399-10172	F9399	TAAGAACAATGAGTTACAAATTGGCAATAG
			R10172	GGGAACACACACTAGCTTACTTCTAAA

SIVsmE543-3

		Nucleotides	Primers	Sequence (5' -> 3')
1	1st	7-1289	F7	GGATTTATTACAATGAGAAAAGACATAGAA
			R1289	TAAATTTTCTGAACCTGTCGGAACTAAT
	2nd	54-1172	F54	GAAAAGGAAGAAGGAATAATACCAGATT
			R1172	CCATACTACATGCTTCAACATATACTTTTT
2	1st	871-2250	F871	TCCTGAGTACGGCTGAGTGAAGGC
			R2250	TCCAACACTTGATTGTCCTCCTTT
	2nd	907-2203	F907	AGGAACAAACCACGACGGAG
			R2203	AATGGGAGTTGGTCTGGTCTCAGT
3	1st	2073-3495	F2073	AAGGGTCTGGGTATGAATCCCACT
			R3495	AATCCTATGCCGTTCAGAAGGTTCC
	2nd	2107-3456	F2107	TGCTGACAGCCTGTCAGGGAATAGG
			R3456	ACCCTGTCATGCTCTAAATCTGTTC
4	1st	3317-4780	F3317	GGGTCACCAGCTATTTTTCAGTAT
			R4780	AAATCCACTAGCCACATGTACTGC
	2nd	3362-4730	F3362	CCTTTCAGAAAAGCAAATCCAGATG
			R4730	GCGTACAGTCCATTTGCCAAGTCCC
5	1st	4594-5945	F4594	ATTGGTATTCAAATTTGGTATACC
			R5945	AGCTCCCTCGGCAGGTGATTTACT
	2nd	4628-5886	F4628	GCAAAACAGATAGTAGACACATGTG
			R5886	CCATTCGAAGGCCTCTTCTATTGTT
6	1st	5721-7136	F5721	AGGGGAGAGAAATTGCTGTCTTGC
			R7136	ACCCTGTCATGTTAAATTTACAAC
	2nd	5754-7095	F5754	CCGAAAGCTCATAAAAATCAGGTAC
			R7095	TCCAAGCCTGCACAACTATTATTTT
7	1st	6719-8416	F6719	ATTCCCCTCTTCTGTGCAACCAGG
			R8416	CTCGATAGCAGTGACTCTAGTCTG
	2nd	6755-8378	F6755	TGGGGAACAACACAATGCTTGCCTG
			R8378	CCCAGACGGTCAGTCGCAACAATTC
8	1st	8257-10039	F8257	CGCGGCGTCGCTGACGCTGTCGGC
			R10039	CTGCCAGCCTCTCCGCAGAGCGAC
	2nd	8290-9988	F8290	GACTTTGTTGGCTGGGATAGTG
			R9988	TTATACAGAGAATAAGTGGGCGTTC
9	1st	8394-10271	F8394	AGACTAGAGTCACTGCTATCGAGAAGTA
			R10271	CTAACAGACCAGGGTCTTCTTTATGT
	2nd	8510-10204	F8510	GTGCCTAATTGGGACAATATGACTT
			R10204	GAACACACACTTGCTTACTTCTAAATG

Primers specific for SIVmac239 and SIVsm543-3 sequence were designed based on GenBank database accession number M33262 and U72748, respectively.
No. of nucleotides described above is counted from the beginning of 5' LTR region.

Table S5. Primers for nested PCR for SIV sequences

Animal	PCR	Nucleotides	1st/2nd	Primers	Sequence (5'→3')
Mm0304	543	8290 - 9250	1st	F8257-543	CGCGGCGTCGCTGACGCTGTCGGC
				R10039-543	CTGCCAGCCTCTCCGCAGAGCGAC
			2nd	F8290-543	GACTTTGTTGGCTGGGATAGTG
				R9250-543	CTCAAGCCCTTGCCTGAC
	543-239	8290 - 9947	1st	F8257-543	CGCGGCGTCGCTGACGCTGTCGGC
				R10004-239	CTGCCAGCCTCTCCGCAGAGCGAC
			2nd	F8290-543	GACTTTGTTGGCTGGGATAGTG
				R9947-239	ATCAAGAAAGTGGGCGTTCCCGACC
Mm0409	543	7916 - 8642	1st	F7842-543	TAGAGGACAGAGACCAAAATAGTAACAGAT
				R8734-543	CCCTACTACTCCTAAAACTATTAGTACACCA
			2nd	F7916-543	CCATGTCATATTAGACAAATAATCAACAC
				R8642-543	TTAGTTTTTGCAATTCATACATATTCTTTT
	543-239	7916 - 8620	1st	F7842-543	TAGAGGACAGAGACCAAAATAGTAACAGAT
				R8712-239	CAGTATTACTCCTACAACTATATAAACTCCA
			2nd	F7916-543	CCATGTCATATTAGACAAATAATCAACAC
				R8620-239	TCAACTTTTGTAATTCATACATGTTCTTCT
Mm0518	543	8955 - 9658	1st	F8870-543	GAAGAAGGAGAAGGTGGAGACA
				R9705-543	ACTGATGAGTCTGTGCTGGATG
			2nd	F8955-543	TACGCCTCTTGACTTGGCTATT
				R9658-543	TGGGCTTCATCTGAGACATCTA
	543-239	8928 - 9375	1st	F8870-543	GAAGAAGGAGAAGGTGGAGACA
				R9444-239	GAAGTTTGAGCTGGATGCATTA
			2nd	F8928-543	ATATTCATTTCCTAATCCGCCA
				R9375-239	ACTAATTTCCATAGCCAGCCAA
Mm0513	543	6560 - 7359	1st	F6494-543	CAGCATTGTTTTCTTAAAAAGGG
				R7396-543	TGAAGAAACCACTACCTTAGAACA
			2nd	F6560-543	AAAGACTAAGACTAATCCACTTCC
				R7359-543	CCTGAATAATTTGAATCATTACACCTAAG
	543	6755 - 8378	1st	F6719-543	ATTCCCCTCTTCTGTGCAACCAGG
				R8416-543	CTCGATAGCAGTGACTCTAGTCTG
			2nd	F6755-543	TGGGGAACAACACAATGCTTGCCTG
				R8378-543	CCCAGACGGTCAGTCGCAACAATTC

Primers specific for SIVmac239 and SIVsm543-3 sequence were designed based on GenBank database accession number M33262 and U72748, respectively. No. of nucleotides described above is counted from the beginning of 5’ LTR region.
